# Revision total knee arthroplasty with long-stem prosthesis following primary knee replacement for giant cell tumor with fracture: a case report

**DOI:** 10.1093/jscr/rjaf789

**Published:** 2025-10-06

**Authors:** Dung A Vu, Nhat D Vu, Tien T Nguyen, Tien T Pham, Ngoc B Thai

**Affiliations:** Department of Joint Surgery, 103 Military Hospital, Hanoi 12108, Vietnam; Department of Orthopaedic and Trauma, Vietnam Military Medical University, Hanoi 12108, Vietnam; Graduate School of Medical Science, Kanazawa Medical University, Ishikawa 920-0293, Japan; Department of Joint Surgery, 103 Military Hospital, Hanoi 12108, Vietnam; Department of Orthopaedic and Trauma, Vietnam Military Medical University, Hanoi 12108, Vietnam; Department of Joint Surgery, 108 Military Central Hospital, Hanoi 11610, Vietnam; Department of Joint Surgery, 103 Military Hospital, Hanoi 12108, Vietnam; Department of Orthopaedic and Trauma, Vietnam Military Medical University, Hanoi 12108, Vietnam; Department of Orthopaedic and Trauma, Vietnam Military Medical University, Hanoi 12108, Vietnam

**Keywords:** giant cell tumor, total knee arthroplasty, revision, long-stem prosthesis, pathological fracture

## Abstract

This case report describes a 30-year-old woman with a rare giant cell tumor (GCT) in the distal femur complicated by a fracture. Initially, treated with total knee arthroplasty, the patient experienced implant loosening after four years, requiring revision surgery with a long-stem prosthesis. The procedure successfully restored joint stability and function, with no tumor recurrence observed at the 18-month follow-up. This case demonstrates the effectiveness of megaprosthetic revision in managing complex GCT-related failures, offering both oncologic control and durable biomechanical reconstruction.

## Introduction

Giant cell tumors (GCTs) are rare, locally aggressive neoplasms that primarily affect the epiphyseal regions of long bones, such as the distal femur or proximal tibia in 55% of cases [1]. Although 80% of these are benign, they can erode bone extensively, resulting in pathological fractures in ~15% of patients [1]. These solitary tumors show are predominantly female and peak in the third decade, with fractures occurring more frequently in older age groups [2, 3].

Fractures through GCTs challenge treatment, reducing the effectiveness of curettage and often requiring prosthetic reconstruction, although recurrence or metastasis rates remain unchanged [4]. Treatment varies from curettage to wide resection, with primary total knee arthroplasty (TKA) used for significant bone loss or fractures to restore function and remove tumor tissue [5, 6]. However, TKA in patients with GCTs is associated with complications such as infection, periprosthetic fractures, and implant loosening due to weakening bone [7]. Revision with a long-stem prosthesis can stabilize the joint by bypassing defective bone [8].

This report describes a patient with a distal femoral GCT complicated by fracture, initially treated with TKA using polymethylmethacrylate (PMMA) for metaphyseal reconstruction. The implant loosened without evidence of tumor recurrence, necessitating revision with a distal femoral megaprosthesis that achieved stable diaphyseal fixation. This case underscores the importance of selecting appropriate metaphyseal reconstruction and fixation strategies, adding insight to the literature on GCT management.

## Case presentation

A 30-year-old woman presented with persistent left knee pain persisting for two years following a primary TKA. She described the pain as moderate to severe, intensifying during weight-bearing activities as walking, which progressively worsened over time.

**Figure 1:**
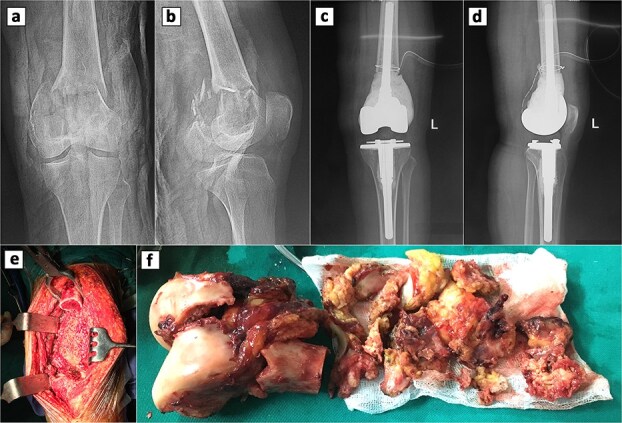
Radiographic and intraoperative findings in primary total knee arthroplasty. Preoperative anteroposterior (a) and lateral (b) radiograph of the knee showing a GCT with an associated fracture in the distal femur. Postoperative anteroposterior (c) and lateral (d) radiograph of the knee following primary total knee replacement. (e) Complete resection of the distal femur. (d) Gross specimens of the distal femur.

**Figure 2:**
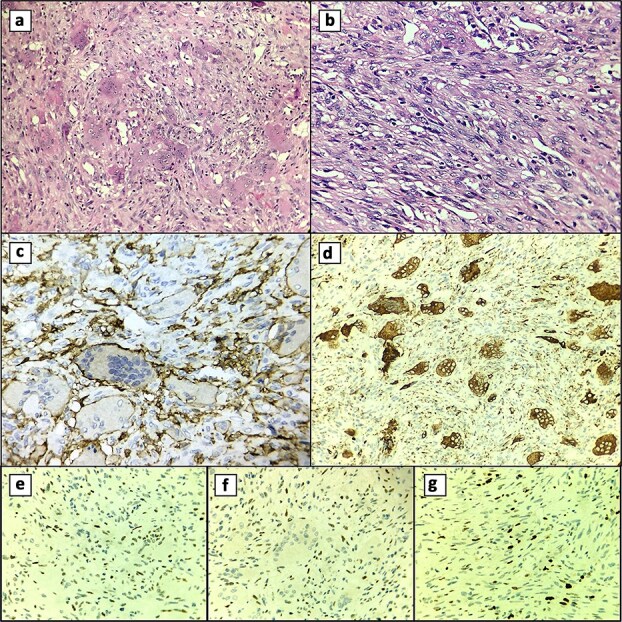
Histological features. (a) Multinucleated giant cell component. (b) Mononuclear cell component. (c) CD45 (+) membranous and cytoplasmic staining of multinucleated giant cells. (d) CD68 (+) cytoplasmic staining of multinucleated giant cells. (e) p53 (+) nuclear staining in mononuclear cells. (f) Nuclear staining in mononuclear cells with p63 (+). (g) Ki-67 (+) in 10% of mononuclear cells.

Four years earlier, she had a pathologic distal femur fracture after a minor fall. Radiographs showed a comminuted metaphyseal fracture with an expansile lytic lesion across both condyles to the articular surfaces, cortical thinning, periosteal lift, and soft-tissue haze (Fig. 1a and b). A ten-year history of dull knee pain implied a chronic lesion with subchondral erosion, leaving no base for fixation or joint salvage. Thus, intralesional curettage and osteosynthesis carried high risks of mechanical failure and local recurrence, especially with articular breach and extensive bone loss. Conversely, segmental resection with primary cemented endoprosthetic TKA allowed en bloc removal, immediate restoration of alignment and congruity, and early full weight-bearing; the stemmed construct bypassed the compromised metaphysis. The patient therefore underwent complete excision of the distal femur and lesion with cemented TKA, achieving a stable reconstruction (Fig. 1c–f). Histology and Immunohistochemistry (IHC) confirmed a GCT composed of multinucleated giant cells and mononuclear stromal cells, supporting the oncologic choice of resection arthroplasty (Fig. 2a–g).

**Figure 3:**
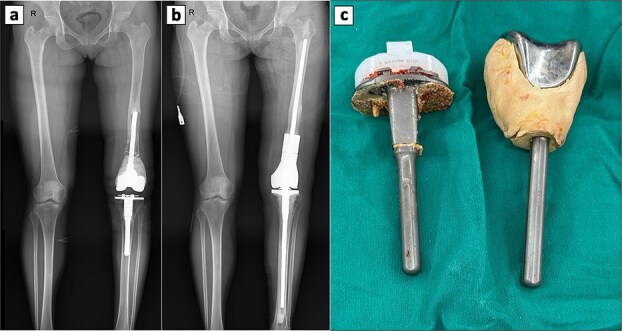
Perioperative radiographic imaging and retrieved prosthetic components following revision total knee arthroplasty. (a) Anteroposterior radiograph after primary total knee replacement, showing the femoral prosthesis loosening. (b) Anteroposterior radiograph after revision total knee replacement. (c) Retrieved prosthesis from the primary total knee arthroplasty, including femoral and tibial components.

Four years later, persistent knee pain prompted further work-up. X-rays showed radiolucent lines around the tibial component, suggesting loosening, while alignment was intact and osteolysis was absent. A revision TKA was performed through the previous scar using a standard anterior approach. Intraoperatively, the femoral cement mantle was loose and the synovium inflamed and hypertrophic, so the original prosthesis was removed (Fig. 3c). Residual cement was cleared, and the tibial component was extracted. Interface tissue demonstrated a foreign-body granulomatous reaction. A megaprosthesis was implanted, and the procedure was completed without issues (Fig. 3a and b). The postoperative course was uneventful; the patient was discharged on day 12, with pain well controlled at the two-week follow-up.

At 18-month post-revision, the patient achieved full weight-bearing status and showed no pain, mechanical issues, or tumor recurrence.

## Discussion

Managing GCTs around the knee requires a careful balance between reliable oncologic control and durable joint mechanics. The distal femur and proximal tibia are subject to high loads and wide ranges of motion, so expansive, lytic metaphyseal disease creates the twin challenges of eradication and stable fixation near the joint line [3]. Plate fixation or intramedullary devices may stabilize pathologic fractures, but constructs anchored in severely compromised periarticular metaphyseal bone often fail, directing attention to prosthetic solutions that permit immediate weight-bearing while preserving motion [9]. Arthroplasty can meet these aims, though long-term data in benign tumors are less robust than in malignant cohorts, and prosthetic complications remain a concern [10, 11].

Options for rebuilding large metaphyseal defects include PMMA, metallic augments (cones/sleeves), and segmental endoprostheses. PMMA offers immediate structural fill and can facilitate adjuvant local control, yet it lacks biological integration and may allow micromotion at the metaphyseal–diaphyseal junction over time. Metallic augments can improve load transfer and achieve osseointegration when host bone is adequate, but performance depends on residual bone stock and may be limited by stress-shielding or loosening in compromised canals. When metaphyseal support is profoundly unreliable, distal femoral endoprosthesis (megaprosthesis) provides predictable mechanics and early mobilization, albeit with risks of infection, aseptic loosening, and mechanical failure; revision rates in oncologic knee reconstructions are substantial, and minimizing diaphyseal resection length (e.g. <30%) may mitigate risk [12, 13].

Across approaches, longevity hinges on secure diaphyseal fixation. Practically, this means a well-fitted, sufficiently long stem that bridges the compromised metaphysis with ample diaphyseal purchase, achieves strong canal fill, and—if cemented—uses firm pressurization to form a uniform, durable mantle. Inadequate diaphyseal purchase allows toggle at the metaphyseal-diaphyseal junction with progressive radiolucency, ending in aseptic loosening despite disease control.

Here, the initial stemmed TKA with PMMA metaphyseal fill prioritized bone preservation in a young patient, early function, and intra-articular resection without diaphyseal sacrifice. Soft-tissue conditions and extensor mechanism integrity favored this approach, and meaningful diaphyseal purchase was anticipated at the time. The subsequent course progressive loosening after four years with no evidence of recurrence implicates mechanical insufficiency of diaphyseal fixation rather than oncologic failure, consistent with the typical timeframe for GCT recurrence declaration [14, 15]. Revision to a distal femoral megaprosthesis addressed these limitations by providing robust diaphyseal fixation and reliable load transfer across the resected segment, restoring alignment, stability, and function while maintaining oncologic safety. This experience underscores three practical points: define and achieve sufficient diaphyseal fixation at the index procedure; recognize that PMMA or metallic augments are viable only when diaphyseal purchase is truly secure; and consider early endoprosthesis when durable metaphyseal support cannot be guaranteed.
